# Estimation of elemental concentrations in the toenail of young Saudi females with obesity

**DOI:** 10.25122/jml-2022-0017

**Published:** 2022-05

**Authors:** Hessah Al-Muzafar, Mohammed Al-Hariri

**Affiliations:** 1. Department of Chemistry, College of Science, Imam Abdulrahman Bin Faisal University, Dammam, Saudi Arabia; 2. Department of Physiology, College of Medicine, Imam Abdulrahman Bin Faisal University, Dammam, Saudi Arabia

**Keywords:** Obesity, females, toenail, Saudi, elements, elemental concentrations

## Abstract

Elemental homeostasis is essential for maintaining normal metabolic processes. Elements in the toenails are now considered in the diagnosis or screening and used as biomarkers of several metabolic disorders. The incidence of obesity is more prevalent in females than males globally. At the same time, females appeared more susceptible to elemental alterations than males. This study aimed to evaluate the variation in the levels of several elements in toenails as possible biomarkers of health conditions associated with obesity in young Saudi females. A cross-sectional study was performed, between February–November 2019. The study enrolled 79 young females divided into two groups: participants with obesity (n=39) and non-obese (n=40). The toenail was analyzed to estimate Fe, I, K, Na, Cd, Cr, Mn, Ca, Mg, Cu, Co, and Se levels. The study showed a significant elevation in the levels of Fe, Ca, K, and Na in the toenail sample of female participants with obesity compared to the non-obese group. The levels of Mn, Cd, Co, Cu, and Cr, were significantly decreased in the toenail of participants with obesity. Moreover, other elements (*i.e*., Mg, I, and Se) were not significantly lower in the female group with obesity. Our findings confirmed the alterations of several elements among Saudi females with obesity. The toenail elemental analysis may become a useful diagnostic technique in monitoring the nutritional status, predicting some metabolic disorders, and environmental exposure.

## INTRODUCTION

Obesity is one of the greatest health challenges worldwide and is associated with the incidence of many disorders such as hyperlipidemia, diabetes, cardiovascular diseases, metabolic syndrome, malignancies, and respiratory illnesses [1]. Alterations in the elemental concentrations, *i.e*., metals and minerals, have been linked with obesity [2]. In Saudi Arabia, the estimated prevalence of obesity among females (≥15) is 36% [3].

Obesity is one of the main risk factors for morbidity and mortality due to non-communicable diseases. It is a preventable and modifiable risk factor for dyslipidemia, metabolic syndrome, and insulin resistance that may lead to disturbances in glycemic control [4].

Elemental homeostasis is essential for maintaining normal metabolic processes. Elements in the toenails are now considered in the diagnosis or screening and used as biomarkers of several metabolic disorders and environmental exposure [5]. Exposure to high levels of certain elements can affect the cell membrane, altering many cellular structures and/or functions such as transporters, enzymes, deoxyribonucleic acid, and signaling systems. In addition, the lack of some elements can compromise the overall health [6]. The nail samples benefit from a “time-integrated measure of exposure and body intake” because of the low growth rate of nails [7]. In addition, toenails are relatively sheltered from external or introduced contaminants through medication, chemicals, and shampooing. Moreover, human nails contain keratin-rich proteins that correlate proportionally based on their serum concentrations with elemental levels [8].

In clinical studies, using nails as markers for element status as a non-invasive specimen may increase the participation rate [9]. Other advantages include collecting and storage, making a study cost-effective [10]. In the same field, the assessment of most elements, 24-hour recall blood test, and dietary frequency questionnaires, cannot appropriately capture the real element concentrations in the body because of their minimal concentration and the continuous body homeostatic control. Thus, using nails to determine the element levels is preferred over other types of analysis [9, 11].

Healthy females have nearly double the amount of subcutaneous fat and fat storage than healthy males [12]. Severe obesity is more prevalent in females than males globally, and obesity-related outcomes are different in females and males [13]. At the same time, females appeared more susceptible to elemental alterations than males [14]. However, results concerning elemental alterations in obesity are still contradictory [15].

There is no reported data available on the elemental concentration in the nail of females in Saudi Arabia. Therefore, we aimed to investigate the variation in elemental levels in the toenails as possible biomarkers of health conditions associated with obesity in young Saudi females.

## MATERIAL AND METHODS

### Participant characteristics

A cross-sectional study was performed between February and November 2019 using convenience sampling and enrolling undergraduate female students at Imam Abdulrahman Bin Faisal University, Saudi Arabia. The eligibility criteria included healthy female participants (who did not have any chronic disease) and non-smokers. The study excluded overweight female participants (body mass index [BMI]=25.0 to <30), pregnant, and with a history of taking a mineral in the last month. The participants were divided into females with obesity (OG) BMI>30 kg/m^2^) and normal, non-obese group (NOG), (BMI=18.5–25 kg/m^2^) [16].

### Determination of element contents in toenail samples

The free edge of the nail from the two great toes was taken by a stainless-steel nail clipper (pretreated with ethyl alcohol) on the same day for each participant. Toenail specimens (5 mg) from each participant were collected in a polyethylene bag either from naturally non-polished or after removing the nail polish before cutting the nails.

All samples of toenails underwent the washing procedure according to the protocol by Blazewicz et al. and Ishak et al. [17, 18]. Each washing stage took 10 min, and after drying in an oven (30 min at 100°C), they were weighed. The weighing stage was performed after washing and drying to prevent possible sample loss. All samples were carefully stored at 25°C [15].

A total of 5 mg of toenail sample was washed in series with H_2_O_2_ (30%), acetone, HNO_3_ (65%), Triton-X 100 (Merck, Darmstadt, Germany). Afterward, the sample digestion and dilution procedures were carried out following the method described by Ishak and his colleagues in 2015 [17]. HNO_3_ (0.5 mL) was added and left overnight at room temperature. The toenail specimens were kept in a drying oven (60°C) for one hour. After cooling, 0.2 mL of H_2_O_2_ was added, and all toenail specimens were incubated again for one hour in a drying oven (60°C). The mixtures were diluted to 10 mL using deionized water [17].

The analyses of the study elements [Iron (Fe), Iodine (I), Potassium (K), Sodium (Na), Cadmium (Cd), Chromium (Cr), Manganese (Mn), Calcium (Ca), Magnesium (Mg), Copper (Cu), Cobalt (Co) and Selenium (Se)] were carried out in the laboratory of Saudi Food & Drug Authority (SFDA) with simultaneous Inductively Coupled Plasma Emission spectrometers (ICPE-9800, ©Shimadzu Corporation, Japan). The spectrometer was optimized daily with a 10µg/L solution of the study elements in a blank solution (1% HNO_3_). The limits of detection were calculated as the level corresponding to three times the standard deviation of ten replicate measurements of the blank solution. All solutions of ICP elements (Merck, Germany) were prepared daily by the dissolution of the reference materials in water and used for calibration. Standards, blanks, and samples were measured with 103Rh as the internal standard (10µg/L, Merck, Germany) [15]. Procedures on quality control were performed daily before and after each set of analyses using the certified reference material (Inductively Coupled Plasma Spectrometer, ©Shimadzu).

### Statistical analysis

Data were analyzed using IBM SPSS, version 24. The normality of continuous variables was assessed using the Shapiro-Wilk test. Mean analysis was performed using the Mann-Whitney U test. Normally distributed data, *i.e*., anthropometric data and age, were presented as mean and standard error of mean (SEM), whereas data with non-parametric distribution were expressed as median and 25–75 percentile boundaries. Statistical significance was considered at p<0.05.

## RESULTS

### Participant characteristics

Descriptive data of the study participants are shown in Table 1. There were 79 participants enrolled in this study, with 39 included in the OG, and 40 in NOG. The study results revealed that the mean BMI of the study participants in OG was 35.4±4.6 kg/m^2^, and for NOG, it was 22.8±1.6 kg/m^2^. The mean age of participants in NOG and OG were 20.5±1.6 and 21.3±1.8 years, respectively.

**Table 1:** Participant characteristics.

	OG	NOG
**Participants (#)**	39	40
**Mean age±SEM**	21.3±1.8	20.5±1.6
**BMI±SEM**	35.4±4.6	22.8±1.6

**OG – obese group; NOG – non obese group; SEM – standard error of mean; BMI – body mass index.Elemental analysis**

**Figure 1:**
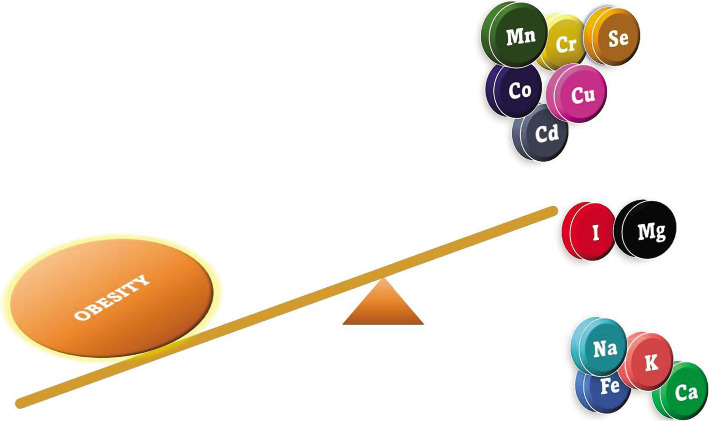
Elemental alteration in the toenails of young Saudi females with obesity.

The levels of Ca (p<0.001), Fe (p<0.001), K (p<0.001), and Na (p<0.001) in the toenail of participants with OG were significantly higher as compared to participants in NOG. In contrast, Cd, Co, Cu, Cr, and Mn levels were significantly lower in the OG than in NOB (p<0.001). Furthermore, the levels of the other studied elements (Mg, I, and Se) were lower in OG as compared to NOG; however, those differences were not significant (Figure 1 and Table 2).

**Table 2:** Comparison of studied elements (mg/kg) in the toenail samples of OG and NOG.

Element	OG Median (Interquartile range)	NOG Median (Interquartile range)	P-value
**Ca**	170.7 (120–240)	54.9 (20–180)	<0.001
**Cd**	1.5 (1.2–2.7)	3.5 (2.1–35.7)	<0.001
**Co**	7.5 (6.7–7.9)	10.1 (8.3–19.6)	<0.001
**Cr**	11.5 (10.7–12.0)	14.9 (8.3–19.6)	<0.001
**Fe**	40.3 (34.4–64.4)	35.9 (29.4–44.4)	<0.001
**I**	4.4 (2.9–7.3)	6.2 (4.4–13.5)	0.205
**K**	220.2 (180.8–260.4)	58.0 (23.2–250.2)	<0.001
**Mg**	28.1 (21.1–36.2)	30.8 (21.1–36.7)	0.963
**Mn**	0.3 (0.5–1.8)	0.7 (0.1–3.2)	<0.001
**Na**	131.0 (98.9–194.0)	39.2 (33.7–111.7)	<0.001
**Se**	8.3 (8.1–13.8)	16.5 (7.7–19.1)	0.190
**Cu**	12.4 (11.9–14.8)	17.6 (14.1–27.1)	<0.001

OG – obese group; NOG – non obese group.

## DISCUSSION

We found high levels of Fe, Ca, Na, and K and low levels of Cr, Cd, Co, Cu, and Mn in the toenail of females with obesity in Saudi Arabia. Alteration in the levels of these elements might play a vital role in the appearance and/or progression of several diseases. For example, reduced or elevated elements (Se, Cu, and Fe) may indicate the presence of depression in young people [19, 20].

The high level of serum ferritin is associated with the accumulation of adipose tissues independent of dietary iron intake [21]. Obesity-associated inflammatory signaling can cause a persistent elevation of blood hepcidin, resulting in decreased intestinal absorption of Fe and erythropoiesis as well as sequestration of Fe in metabolic tissues [22, 23], leading to subsequent accumulation of Fe in body tissue [24], including nails [25]. Therefore, these factors could explain our findings regarding the significant elevation of Fe in the toenails of females with obesity compared to the non-obese group.

In this study, the obese females showed a higher level of Ca than the non-obese females. Previous studies suggested some explanations for this metabolic disruption, including volumetric dilutional, physiological sequestration of vitamin D in adipose organs, adaptation to the need for more bone mass to support weight gain [26], and secondary hyperparathyroidism [27]. At the same point, obesity is believed to induce the production of inflammatory biomarkers that stimulate bone uptake by osteoclasts and may subsequently lead to higher serum Ca levels in obese individuals, which may, in turn, result in the mobilization of Ca from bone to other body compartments [28].

This study revealed a significant increase in the level of Na in the toenail of females with obesity. Earlier data reported an anomaly in the hormone mechanism associated with Na homeostasis that may contribute to the development of hypertension and hypernatremia in participants with obesity [29]. A previous review has explained the role of obesity-related hyperinsulinemia in Na retention. In this regard, insulin may increase the absorption of Na in the renal tubules and might increase Na accumulation [30].

In addition, the level of K in the toenail was significantly much higher among females with obesity than those in the NOG. K is an intracellular element necessary for the normal function of all living cells. Many researchers have investigated the associations between K and obesity. However, these observational results are considered controversial [30]. This may be explained partially by the contentious homeostatic regulation control of serum K. While blood and urine tend to show recent or current body status, the toenail sample may help assess a longer time frame of K level [30, 31]. However, the total body K level is directly associated with the body cell mass [32].

In this study, Cr, Cd, Co, and Mn were significantly lower in OG participants. These data are in accordance with earlier experimental reports [33, 34]. These findings encourage further studies concerning the use of biological sample as a biomarker of element status [35]. Obesity is associated with low-grade chronic inflammation, which can produce a variety of adipocytokines, [36] being positively correlated with element alteration [37]. Although elements in biological samples have been studied for many decades, the existing data are insufficient, and the effect of interactions on obesity is not often studied in practice [38].

It was reported that blood Cr was reduced in obese participants. Vice versa, Cr supplementation could be a promising agent in modulating obesity [38]. However, existing data on Cr status in obese participants are insufficient [39]. The existing data on the relationship between obesity and Cd are anecdotal [34]. Certain reports found a negative association between Cd and anthropometric indices of obesity [40]. Oppositely, others failed to detect any significant relationship between the bodyweight and Cd level [41].

Unfortunately, little is known about the effect of Co level on obesity in general. In the present study, the Co level in toenails from the OB group was lower than in NOB. The same finding was observed in another study using a plasma sample [38].

There is evidence of direct interaction between the elements. *i.e*., “deficiency in one element may impair the absorption of another”, [42] which could be the possible cause of the lower Cu toenail content observed in our study. The observation that lower Mn levels in the toenails of females with obesity was consistent with our previous study on Mn levels in a different biological sample [43].

The lower level of Se in the toenail of females with obesity was in agreement with a previous report that showed an inverse association between obesity and Se levels in nails [44]. However, the statistical analysis revealed no significant difference at the median level of Se in both groups. Perhaps this could be due to the relatively small size of the sample [45].

Moreover, despite the absence of statistically significant differences, the observed decrease in levels of Mg and I are generally consistent with findings from previous studies where it was reported that obesity increases the incidence of Mg [46] and I deficiency [47].

## CONCLUSION

The present data added new information on the elements from the toenails of young Saudi females with obesity. The elemental analysis of the nail may become a useful diagnostic technique in monitoring nutritional status, predicting some metabolic disorders, and environmental exposure in the future. However, further studies are needed with a representative sample size to precisely elaborate reference ranges of elements in the nail and formulate universal guidance on methodology considering age, gender, and lifestyle. The medical care plan for obese individuals should include behavioral counseling to reduce their exposure to high-calorie foods with low nutritional value. To the best of our knowledge, this is the first study that reports elemental alterations in the toenail of females with obesity in Saudi Arabia. Expert lab technicians used a very advanced automated instrument for the elemental analysis in a qualified and specialized governmental institute (SFDA).

There were some limitations in our study. Firstly, the study did not correlate the results with demographic variables of other health and lifestyle data of the study sample, which are essential in future data collection. Secondly, there was a small sample size, and the focus was on only one gender and a specific age group. Third, we did not correlate the elemental levels in toenails with blood levels.
